# Mitogenomics of the tropical bont tick *Amblyomma variegatum* reveals vertical and horizontal transmission of *Rickettsia africae*

**DOI:** 10.1371/journal.pntd.0013610

**Published:** 2025-10-21

**Authors:** Elisha Chatanga, Wessam Mohamed Ahmed Mohamed, Samuel Kelava, Naoki Hayashi, Yuma Ohari, Mohamed Abdallah Mohamed Moustafa, Joseph W. Magona, Kyoko Hayashida, Yongjin Qiu, Nariaki Nonaka, Ryo Nakao

**Affiliations:** 1 Department of Veterinary Pathobiology, Faculty of Veterinary Medicine, Lilongwe University of Agriculture and Natural Resources, Lilongwe, Malawi; 2 Laboratory of Parasitology, Graduate School of Infectious Diseases, Faculty of Veterinary Medicine, Hokkaido University, Sapporo, Japan; 3 Department of Molecular Biology, Princeton University, Princeton, New Jersey, United States of America; 4 Division of Parasitology, Veterinary Research Unit, International Institute for Zoonosis Control, Hokkaido University, Sapporo, Japan; 5 Division of Risk Analysis, International Institute for Zoonosis Control, Hokkaido University, Sapporo, Japan; 6 One Health Research Center, Hokkaido University, Sapporo, Japan; 7 Department of Animal Medicine, Faculty of Veterinary Medicine, South Valley University, Qena, Egypt; 8 Department of Diagnostic Medicine and Pathobiology, College of Veterinary Medicine, Kansas State University, Manhattan, Kansas, United States of America; 9 Africa Union, Interafrican Bureau for Animal Resources (AU-IBAR), Nairobi, Kenya; 10 Division of Collaboration and Education, International Institute for Zoonosis Control, Hokkaido University, Sapporo, Japan; Kenya Agricultural and Livestock Research Organization, KENYA

## Abstract

**Background:**

The tropical bont tick *Amblyomma variegatum,* which is widespread in Africa and the Caribbean islands, is of both medical and veterinary importance as the principal vector of intracellular bacterial pathogens *Ehrlichia ruminantium*, causing heartwater in animals, and *Rickettsia africae*, causing African tick bite fever (ATBF) in humans. This tick species is highly invasive and has been reported to expand its geographical distribution as well as host range. *Rickettsia africae* is also recognized as a common endosymbiont in *A. variegatum*, but its transmission dynamics within this tick population remain poorly understood.

**Methodology:**

To investigate the co-phylogenetic patterns between *A. variegatum* and *R. africae*, we sequenced the complete mitogenomes of *A. variegatum* and performed multi-locus sequence typing (MLST) of six housekeeping genes of *R. africae*. The resulting sequence data were used to examine the hypothesis that *R. africae* is predominantly transmitted vertically within *A. variegatum* populations, which would lead to congruent phylogenies between vector and pathogen.

**Results:**

There was geographical population sub-structuring in the mitogenomes of *A. variegatum*. The prevalence of *R. africae* in the examined ticks was 100%. The tanglegram showed non-strict co-cladogenesis between *A. variegatum* and *R. africae*. Furthermore, the Procrustes Application to Cophylogenetic (PACo) analysis and residuals of vector-pathogen associations showed no statistically significant association between *A. variegatum* and *R. africae* genotypes.

**Conclusions:**

This study was the first to examine the spread of pathogenic/endosymbiotic bacterium *R. africae* in the *A. variegatum* populations using a mitogenomic approach. The results support both vertical and horizontal transmission of *R. africae* within *A. variegatum*. These findings also highlight the potential of *R. africae* to adapt to multiple animal species, which may complicate efforts to control it as a human pathogen.

## Authors summary

Investigating the co-phylogenetic relationship between the *Rickettsia africae* and its vector tick, *Amblyomma variegatum*, is essential for understanding the interactions that drive bacterial persistence within the tick and facilitate transmission to other animal species. The ability of *R. africae* to establish itself as a symbiont in *A. variegatum* without compromising tick fitness, while also being pathogenic in humans, highlights its complex and informative biology. This dual role makes it a neglected yet widespread pathogen in Africa, deserving further investigation. This research also provides insight into how *Rickettsia* species establish themselves in ticks. Understanding this interaction is the prerequisite to developing novel methods for the control of tick-borne rickettsioses. This study compared the evolutionary relationships between *A. variegatum* and *R. africae*. The evolutionary histories of the tick and the rickettsial bacterium are apparently not closely linked, as their genetic lineages do not mirror each other. This suggests that the bacteria are not just passed down from parent ticks to their offspring through vertical transmission (which would show matching patterns), but can also spread between different tick individuals through horizontal transmission. This likely happens when ticks feed on the same animals. In short, the bacteria seem to move between different tick populations, rather than being restricted to certain genetic lines of ticks. This makes *R. africae* a more flexible and adaptable organism, which could make it harder to control as a human pathogen.

## 1. Introduction

*Amblyomma variegatum* Fabricius 1794, (Acari: Ixodidae), also known as tropical bont tick, is an important tick of both medical and veterinary significance [1,2]. This invasive tick species is widely distributed in Africa and the Caribbean islands [3,4], but has also been reported in some European countries such as Italy and France [1,2]. *Amblyomma variegatum* is a three-host tick with the larvae, nymphs, and adults each feeding on separate hosts, primarily the Bovidae family [3]. The tropical bont tick serves as a vector for several bacterial, protozoal, and viral pathogens. These include *Ehrlichia ruminantium* and *Anaplasma bovis*, which cause ehrlichiosis and anaplasmosis in ruminants, respectively [3]. It also transmits *Theileria mutans* and *Theileria velifera*, both associated with benign bovine theileriosis [3], as well as *Babesia caballi* [5] and *Babesia* spp. [6]. Moreover, the tick is a vector for several viruses, including Karukera tick virus, Wuhan tick virus 2, Lihan tick virus, and Jingmen tick virus [7].

The tick endosymbiont *Rickettsia africae* has been reported with high prevalence in *A. variegatum* in Africa [8,9]. *Rickettsia* endosymbionts in ticks such as *R. africae* in *A. variegatum* contribute to enhancing tick fitness [10,11]. The tick microbiome has been shown to affect the capacity of ticks to function as vectors by modulating the pathogen colonization within tick tissues [11,12]. Macaluso et al. [13] suggested that tick ovaries, once colonized by one *Rickettsia*, alter the expression of certain genes in the oocytes, rendering them impervious to a subsequent infection by other *Rickettsia*. This has been supported by an experimental study that showed ticks can only be infected with one *Rickettsia* species at a time [14]. The vertical transmission of *R. africae* facilitates its amplification and maintenance in the *Amblyomma* species, as its colonization of the ovaries prevents subsequent infection by other *Rickettsia* species [13].

As a pathogen, *R. africae* is the causative agent of African tick bite fever (ATBF). Initially, it was considered less pathogenic in humans [15] and genetically homogeneous [16]. However, recently reported cases have shown that it has become more virulent with a more pronounced clinical presentation in humans, such as headache, fever, eschars, rash, lymphadenopathy, myalgia, chills, malaise, and arthralgia [15,17,18]. In some cases, complications have included myocarditis, pericarditis, conjunctivitis, floaters, and neurological symptoms, particularly in individuals with underlying conditions such as a history of surgery, asthma, hypertension, diabetes or other co-infections like leishmaniasis [18,19]. Most reported cases of ATBF were from returning residents of Europe, America, and Asia with a history of recent travel to endemic areas in sub-Saharan Africa [20,21]. The non-specific symptoms of ATBF increase the likelihood of misdiagnosis with other endemic diseases in sub-Saharan Africa, such as malaria [22]. Additionally, limited epidemiological data and insufficient diagnostic capacity in the region further hinder accurate detection [23].

The principal vector ticks of *R. africae* are *Amblyomma hebraeum* and *A. variegatum* [3]. However, this bacterium has also been detected in ticks of the genera *Haemaphysalis*, *Hyalomma,* and *Rhipicephalus* [24,25]. *Rickettsia africae* has been detected in both domestic and wild animals, including cattle, donkeys, goats, and sheep among domestic species, and buffaloes, elands, giraffes, and kudus among wildlife [3,26,27]. Other reported vertebrate species include lizards, small mammals, and birds [28]. Humans, however, can be incidentally infected by this bacterium, typically due to zoonotic spillover following bites from infected ticks, often occurring during visits to tick infested wildlife reserves or resorts [15,17,18,20,21].

Although studies have been conducted to understand the genomics, pathogenicity, vector competence, and transmission of *Rickettsia* species [4,6,13,14,17], not much has been done to investigate the co-phylogenetic relationship of *Rickettsia* species within their vector ticks. Investigation of the co-phylogenetic relationship between *R. africae* and *A. variegatum* is important in understanding *Rickettsia*-tick-vertebrate species interactions mediating successful maintenance, disease transmission, pathogenesis, and how *Rickettsia* species establish themselves in ticks and vertebrate species to cause rickettsioses [29–30]. Further, understanding this interaction is the prerequisite to developing novel methods in the control of tick-borne rickettsioses through the manipulation of the tick symbionts or microbiome [30].

To investigate the co-phylogenetic relationship between the bacterial endosymbiont *R. africae* and its principal vector, *A. variegatum*, we employed a mitochondrial genome (mitogenome)-based approach. The mitogenomes of ticks are being used to investigate the evolution, molecular evolution, phylogenetic relationships, systematics, and the identification of cryptic tick species [31,32]. In this study, the complete mitogenome sequences from *A. variegatum* ticks collected from cattle in Malawi and Uganda and the multi-locus sequence typing (MLST) of six genes of *R. africae* were used to test the hypothesis that *R. africae* is predominantly transmitted vertically within *A. variegatum* populations, which would lead to congruent phylogenies between vector and pathogen.

## 2. Materials and methods

### 2.1. Ethics statements

All the procedures were conducted in accordance with the guidelines of Hokkaido University, Japan, for research ethics. The local ethical clearance of the protocol to sample ticks from cattle was approved by the Department of Animal Health and Livestock Development Animal Health and Research Ethics Committee in Malawi (reference numbers DAHLD/AHC/01/2018–001). Verbal informed consent was sought from the animal owners before tick sampling.

### 2.2. Sample collection and DNA extraction

**Fig 1:**
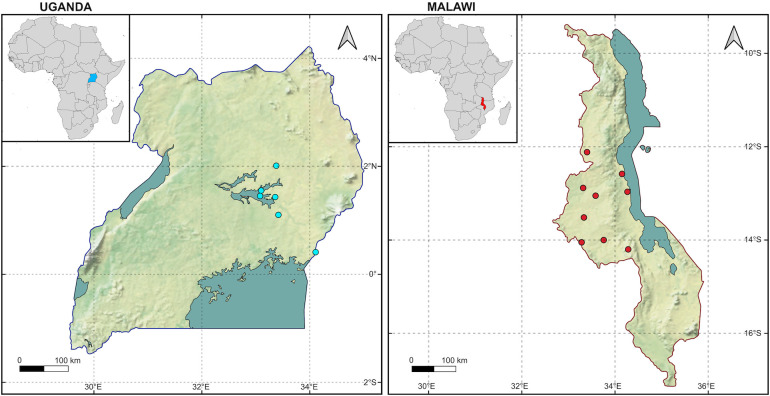
Geographic distribution of *Amblyomma variegatum* samples used in the present study. Samples were collected from nine locations in Malawi and seven districts in Uganda. The base layer of the map was made with Natural Earth (naturalearthdata.com) using the following links: https://www.naturalearthdata.com/downloads/10m-cultural-vectors/10m-admin-0-countries/; https://www.naturalearthdata.com/downloads/10m-physical-vectors/10m-lakes/; https://www.naturalearthdata.com/downloads/10m-natural-earth-1/natural-earth-1-with-shaded-relief/.

Tick samples used in this study were collected from two countries in Africa; Malawi and Uganda, as shown in Fig 1. The DNA of tick samples from Uganda was obtained from a previous study [33]. In brief, the tick sampling in Uganda was done from July 2008 to January 2009 from a local breed of cattle in seven districts. The collected ticks were stored in sealed plastic bags containing silica gel until required for DNA extraction. The sample collection of the ticks was done by picking them directly from the animal’s body using forceps or a tick twister gently to ensure that the mouthparts were intact. Tick samples from Malawi were collected from cattle between January 2018 and March 2019. Collected ticks were preserved in 50 mL centrifuge tubes containing dry silica as described previously by Dillon et al. [34]. The ticks were identified at the species level morphologically under a stereomicroscope using established keys [3,35]. Those ticks that were not engorged were used for DNA extraction using DNAzol genomic DNA isolation reagent (Molecular Research Center, OH, USA) according to the manufacturer’s recommendations. The extracted DNA which averaged around 20 ng/µL was kept at -20°C until required for use.

### 2.3. Long-range PCR amplification for tick mitogenome sequencing

The complete mitogenome of *A. variegatum* was amplified in three overlapping fragments with expected sizes of 7,381 bp (fragment 1), 5,561 bp (fragment 2), and 5,347 bp (fragment 3). Together, these three fragments covered the complete mitogenome of *A. variegatum* of approximately 14–15 kb in length. The mitogenome sequences of *A. hebraeum* obtained from the GenBank (accession numbers KY457512 and KY457513) were used to design primers for the three overlapping fragments. The primers were designed using a modified version of Primer3 v.2.3.7. [36] in the software Geneious Prime (https://www.geneious.com) according to standard protocols [37]. The primers used in this study are listed in Table 1 [38–44].

**Table 1:** Primers and probe used for amplification and sequencing of *Amblyomma variegatum* mitogenome and multi-locus sequence typing of *Rickettsia africae.*

Primer name	Primer sequence (5′ to 3′)	Target organism/gene	Product size (bp)	Reference
Amb.var_long01e	TGCTTTCTCTTGGAGGAATACC	*A. variegatum* mitogenome	~ 7,631	This study
Amb.var_long02e	GCTCTGTAATGGATAAATCGCC			
Amb.var_long03b	CGGTCTAAACTCAGATCATGTAGG	*A. variegatum* mitogenome	~ 5,561	This study
Amb.var_long04c	TTGTGTGTGAAGCTGCATTAGG			
Amb.var_long05c	TCCCGAAATTGGAGCTTCTAC	A.*variegatum* mitogenome	~ 5,347	This study
Amb.var_long06c	AACAGCTCCTATTGACAGAACG			
CS-F	TCGCAAATGTTCACGGTACTTT	*Rickettsia* citrate synthase gene (*gltA*)	74	Stenos et al. [49]
CS-R	TCGTGCATTTCTTTCCATTGTG			
CS-P	TGCAATAGCAAGAACCGTAGGCTGGATG			
gltA_Fc	CGAACTTACCGCTATTAGAATG	*Rickettsia* citrate synthase gene (*gltA*)	580	Gaowa et al. [50]
gltA_Rc	CTTTAAGAGCGATAGCTTCAAG			
Rr.190.70p	ATGGCGAATATTTCTCCAAAA	*Rickettsia* outer membrane A gene (*ompA*)	542	Regnery et al. [51]
Rr.190.602n	AGTGCAGCATTCGCTCCCCCT			
120_2788	AAACAATAATCAAGGTACTGT	*Rickettsia* outer membrane B gene (*ompB*)	816	Roux and Raoult [52]
120_3599	TACTTCCGGTTACAGCAAAGT			
17k_5	GCTTTACAAAATTCTAAAAACCATATA	*Rickettsia* 17-kDa common antigen gene (*htrA*)	550	Labruna et al. [53]
17k_3	TGTCTATCAATTCACAACTTGCC			
Rick_16S_F3	ATCAGTACGGAATAACTTTTA	*Rickettsia* 16S ribosomal RNA gene (16S rDNA)	1,328	Anstead et al. [54]
Rick_16S_F4	TGCCTCTTGCGTTAGCTCAC			
D1f	ATGAGTAAAGACGGTAACCT	*Rickettsia* surface cell antigen-4 gene (*sca4*)	928	Sekeyova et al. [55]
D928r	AAGCTATTGCGTCATCTCCG			

Each reaction was carried out in a 50 μL reaction mixture containing 1.0 µL of Tks Gflex Polymerase (Takara Bio Inc., Shiga, Japan), 25.0 μL of Tks Gflex Polymerase reaction buffer, 10 ng of DNA template, 1.0 µL of each primer (10 µM), and molecular grade water. A negative control containing molecular grade water instead of a tick genomic DNA template was included for quality control.

The PCR conditions were set with the initial denaturation at 94°C for 1 min, followed by 45 cycles of denaturation at 98°C for 10 sec, annealing at 60°C for 15 sec, and extension at 68°C for 7 min. The final extension step was carried out at 68°C for 5 min. Then, 5 μL of the PCR product was used for electrophoresis at 100 V for 45 min in 1% agarose gel stained with Gel-Red (Biotium, Hayward, CA, USA) and visualized under UV light. The remaining PCR product (45 µL) was purified using Nucleospin Gel & PCR clean-up (Macherey-Nagel, Düren, Germany) following the manufacturer’s protocol and DNA was quantified using a Qubit 3.0 Fluorometer (Thermo Fisher Scientific, USA).

### 2.4. Illumina sequencing and data analysis for tick mitogenome construction

Before library preparation, the quantified PCR products were diluted using molecular-grade water to 0.2 ng/μL of DNA. Then the diluted PCR products of the three overlapping fragments from each sample were mixed in equimolar ratios in one tube. Libraries were constructed for each sample using the Nextera XT DNA Library Prep Kit (Illumina, San Diego, CA, USA) and the Nextera XT DNA Library Preparation Index Kit v2 (Illumina), following the protocols of the manufacturer. The DNA of each library was quantified and diluted to 4 nM before pooling. After pooling the DNA was also quantified to ensure that the concentration was within 4 nM range. The pooled multiplexed libraries were sequenced on an Illumina MiSeq platform with the MiSeq Reagent Kit v2 (600 cycles).

We used FastQ [45] to assess the quality of the raw sequence data and the BBDuk plug-in in Geneious Prime for filtering out sequences shorter than 50 bp, trimming low-quality bases (Q-score <25) at the end of reads, and adaptor removal. The trimmed and filtered reads (~485,000 reads for each sample) were mapped to the *A. hebraeum* mitogenome sequence [31] using the Highest Sensitivity/Medium Geneious Mapper Algorithm with up to 25 iterations. Contig sequences were obtained and assembled into circular molecules. The reads were also *de novo* assembled by the software Geneious Prime and CLC Genomics Workbench v21.0.3 (https://digitalinsights.qiagen.com) using default parameters. The trimmed sequence reads were then remapped to the *de novo* assemblies using the software Geneious Prime to ensure coverage and completeness. The contigs were aligned with those generated by mapping the reads to *A. hebraeum* to confirm that the assembly method did not influence the final mitogenome sequences of *A. variegatum*. Gene annotations were performed based on the *A. hebraeum* mitogenome. Transfer RNA (tRNA) genes were confirmed using the software tRNAscan-SE [46] and MITOS [47].

The gene borders were also checked using MITOS and manually curated. The graphical mitogenome map of *A. variegatum* was drawn using the software CGView Server [48]. AT- and GC- skew analyses were calculated following the formulas (A − T)/ (A + T) and (G − C)/ (G + C), respectively [49]. Nucleotide and translation statistics and relative synonymous codon usage (RSCU) were determined using the software programs Geneious Prime and MEGA 7 [50].

### 2.5. Tick phylogeny and network analysis

The complete mitogenome sequences obtained in this study were imported to Geneious Prime and aligned with the mitogenome sequence of *A. hebraeum* available in the database. To understand the intra/inter-species genetic relationship of *A. variegatum*, the phylogeny of *A. variegatum* collected from different geographical locations was examined. Mitogenome sequences of *A. variegatum* from Malawi and Uganda were aligned using MAFFT software [51]. The substitution model was selected using PHYML 3.0 software relying on the Akaike information criterion (AIC) [52]. A pairwise identity analysis was conducted by calculating the percentage of residues that are identical between sequences in Geneious Prime.

Maximum clade credibility tree generated with entire mitogenomes using BEAST v1.4. program as a cross-platform program for Bayesian analysis of molecular sequences using Markov Chain Monte Carlo (MCMC). We modeled the sequence evolution by using the GTR nucleotide substitution model with discrete gamma-distributed rate variation and assuming a constant evolution rate all over the tree by selecting the strict clock model. We assume the Bayesian skyline coalescent model as a demographic model in a Bayesian framework. The Bayesian skyline plot analysis was performed using a chain length of 50 million generations sampled every 50,000 MCMC steps with a pre-burn-in of 500,000. Maximum clade credibility (MCC) tree was selected by using the TreeAnnotator [53] and was illustrated using FigTree v1.4.4 software (http://beast.bio.ed.ac.uk/figtree). The branch length of the tree was transformed to proportional and supported by the posterior values. The median joining network (MJN) was constructed using PopArt software v1.7 (https://popart.maths.otago.ac.nz) [54].

### 2.6. Tick spatial and demographic evolutionary dynamics analyses

The historical expansion dynamics of *A. variegatum* populations were estimated by calculating the mismatch distribution based on complete mitochondrial genomes in Arlequin. We compared the observed and expected distributions of pairwise nucleotide variations between individuals. Multimodal distribution is thought to be associated with a constant population size, whereas unimodal distribution represents a sudden expansion. Departure from the neutral model of evolution was evaluated by calculating Tajima’s *D* [55] statistics in the Arlequin based on the infinite-size model by quantifying the difference between the average pairwise nucleotide differences and polymorphic sites. Mantel test was used to test whether there is a geographic separation between the *A. variegatum* populations from different geographic origins and to evaluate the correlation between genetic variation and geographic distance of the tick population. R function ‘mantel.rtest’ in ape package was used [56]. A scatter plot of the correlation was created using the R package ggplot2 [57,58].

### 2.7. Real-time PCR for detection of *Rickettsia*

The *A. variegatum* samples from which we obtained complete mitogenome sequences were screened for *Rickettsia* using citrate synthase (*gltA*) gene real-time PCR as previously described [33]. The primer and probe information used in this study are indicated in Table 1. The reaction was carried out in a 20.0 μL reaction mixture containing 10.0 μL of THUNDERBIRD Probe qPCR Mix (Toyobo, Osaka, Japan), 300 nM of each primer, 200 nM of probe, 5.0 μL of template DNA, and distilled water. The reaction was carried out in a QuantStudio 12K Flex Real-Time PCR System (Life Technologies Corporation, CA, USA) at the following conditions 50 °C for 3 min, 95 °C for 1 min, and 40 cycles of 95 °C for 15 sec and 60 °C for 1min. Each run included a blank control and serially diluted *Rickettsia asiatica* plasmid standards of (10^6^, 10^4^, and 10^2^ copies/reaction) as previously described [33]. Molecular grade water instead of genomic DNA was used as a negative control for quality control.

### 2.8. Genotyping of *Rickettsia africae* using conventional PCRs and Sanger sequencing

The samples that were positive for *Rickettsia* by real-time PCR were subjected to conventional PCRs for MLST of six genes namely: *gltA* gene, outer membrane protein A gene (*ompA*), outer membrane protein B gene (*ompB*), 17-kDa common antigen gene (*htrA*), surface cell antigen-4 gene (*sca4*), and 16S ribosomal RNA gene (16S rDNA) using the primers listed in Table 1. The reaction was carried out in a 10.0 µL reaction mixture containing 0.2 µL of Tks Gflex Polymerase, 5.0 µL of 2X Tks Gflex Polymerase reaction buffer, 5.0 ng DNA template, 0.5 µL of each primer (10 µM), and molecular grade water. DNA from *Rickettsia asiatica* was used as a positive control while distilled water was used as a negative control for quality control. The PCR conditions were set at initial denaturation at 94°C for 1 min, followed by 40 cycles of denaturation at 98°C for 30 sec, annealing temperature for 30 sec, extension at 68°C for 1 min, and final extension at 68°C for 5 min. The amplicons were visualized as mentioned above.

The PCR products from *R. africae* were purified using ExoSAP-IT (Thermo Fisher Scientific). The amplicons were sequenced in both directions using primers used in conventional PCR (Table 1). Sequencing reactions were performed in a 10.0 µL reaction mixture containing 1.00 ng of purified PCR product, 1.75 µL of 5 × Sequencing buffer (Applied Biosystems, Foster City, CA, USA), 0.50 µL of BigDye Terminator version 3.1 Cycle Sequencing Kit (Applied Biosystems), 0.32 µL of primer (10 µM), and 6.43 µL of distilled water. The sequencing products were purified using Agencourt AMPure XP beads (Beckman Coulter, Brea, CA, USA) and sequenced on 3135 xL Genetic Analyzer (Applied Biosystems).

Sequences were edited using GENETYX version 9.1 (GENETYX Corporation, Tokyo, Japan) to remove the primer annealing sites. The number and percentage of variable sites were calculated using DnaSP v6 [59]. The phylogenetic tree using concatenated sequences of the six genes was constructed in MEGA 7 using the maximum likelihood method with the Kimura 2-parameter model. To test for confidence, the bootstrap values were calculated using 1,000 replications.

### 2.9. Tick and symbiont population genetic structure analyses

The Analysis of Molecular Variance (AMOVA) program in the Arlequin v3.5.2.2 [60] was used to evaluate the genetic variance among and within the populations of *A. variegatum*, and their *R. africae* symbionts, collected from Malawi and Uganda. We set the number of permutations at 1,000 and the difference was significant when tested at P < 0.05 level based on the calculated fixation indices (F_ST_). F_ST_ estimates the degree of differentiation within the population where the closer F_ST_ is to 0, the greater the extent of allelic fixation or identity within populations [61]. Finite Sites Correction (F_SC_) estimates the differentiation among populations within the group to which they are assigned. The closer F_SC_ is to 1, the more heterogeneity among populations exists. In case a strong population genetic structure exists at the population scale being analyzed, F_SC_ should be high relative to F_ST_. To estimate the statistical power of the AMOVA used to detect population structure in *A. variegatum* and *R. africae*, we implemented a permutation-based power analysis in R v4.5.1), using the pegas and ade4 packages. We simulated 1,000 null datasets by randomizing population labels among samples and recalculating Φ_ST_ for each iteration to generate a null distribution. We then calculated the proportion of null Φ_ST_ values less than the observed Φ_ST_ = 0.3111. The statistical power was defined as the proportion of permutation datasets yielding a Φ_ST_ equal to or greater than this observed value.

### 2.10. *Amblyomma variegatum* and *Rickettsia africae* co-phylogeny

To explore the co-phylogenetic relationship between *A. variegatum* and *R. africae*, the phylogenetic trees of the tick (vector) and bacterium (pathogen) were compared using a tanglegram generated in Dendroscope software [62]. Further, to assess the co-phylogenetic relationship between *A. variegatum* and *R. africae*, we conducted a Procrustes analysis using distance matrices derived from vector and pathogen phylogenies using the PACo R package [63]. The analysis involved superimposing the principal coordinate representations of these distance matrices and quantifying their degree of alignment. The results were visualized in a Procrustes superimposition plot, where arrows connect corresponding vector and pathogen taxa. The length of each arrow represents the degree of phylogenetic incongruence, with shorter arrows indicating greater congruence, consistent with vertical transmission, whereas longer arrows indicate weaker congruence and are consistent with horizontal transmission [64].

### 2.11. Nucleotide sequence accession numbers

The complete mitogenome sequences of 41 *A. variegatum* were deposited in the DNA Data Bank of Japan (DDBJ) (http://www.ddbj.nig.ac.jp) with accession numbers of LC834168 - LC834187 (samples from Malawi) and LC834188 - LC834208 (samples from Uganda). The *Rickettsia* sequences obtained in this study were submitted to the DDBJ under accession numbers LC780785 to LC780825 for *gltA* gene; LC780826 to LC780864 and LC782227 to LC782228 for *htrA* gene; LC780865 to LC780905 *for ompA* gene; LC780906 to LC780946 for *ompB* gene; LC780947 to LC780987 for *sca4* gene, and LC782229 to LC782269 for 16S rDNA.

## 3. Results

### 3.1. Tick mitogenome features, phylogeny, and network analysis

The obtained complete mitogenome sequences of the 41 *A. variegatum* ticks ranged from 14,630 bp to 14,651 bp in length. A total of 37 encoded genes were detected including 13 protein-coding (PCGs), 22 tRNA, and 2 ribosomal RNA (rRNA) genes. Two control regions (CRs) were also identified in the mitogenome. The gene arrangement was conserved, as in other Metastriata tick species. The nucleotide sequence of the *A. variegatum* mitogenome contains approximately 39.0% thymine, 37.8% adenine, 13.4% cytosine, and 9.8% guanine. A + T and G + C contents of the complete mitogenome were 76.8% and 23.2%, respectively. Guanine had the lowest frequency and, similar to the content of other known Metastriata tick mitogenomes, the total A + T content across the genome was very high [65]. The A + T frequency of the CRs was lower (66.1%) than that of the complete mitogenome, PCGs (A + T = 76.9%), rRNAs (80.7%), and tRNAs (78.1%) (S1 Table). AT-skew ranged from − 0.22 for *cox3* and *nad3* to 0.24 for *nad4l*), while GC-skew ranged from − 0.5 for *atp8* to 0.03 for *nad3*. The mean AT- and GC- skews were − 0.03 and − 0.2, respectively. The negative values of AT- and GC- skews indicate T and C-biased nucleotide compositions, respectively. These patterns are similar to other tick species [66,67]. The analysis of the PCGs showed that those with high polymorphic sites were *atp8*, *nad6*, *nad2*, and *nad4*, with rates of 8.8%, 7.7%, 7.4%, and 6.8%, respectively (Table 2). In terms of the number of haplotypes among the 41 sequences, *nad4*, *nad5*, *cox1*, *cytb*, and *nad2* had the highest frequencies at 28, 27, 26, 21, and 20, respectively.

**Table 2:** Comparison of mitochondrial gene variations from 41 *Amblyomma variegatum* ticks.

Gene	Nucleotide length (bp)	No. of haplotypes	GC content (%)	Polymorphic sites (%)	Parsimony informative sites (%)	Haplotype diversity ± SD	Nucleotide diversity ± SD
*nad2*	963	20	19	7.4	5.8	0.951 ± 0.015	0.01576 ± 0.00224
*cox1*	1,539	26	30.5	6	4.5	0.951 ± 0.020	0.01222 ± 0.00181
*cox2*	675	10	27.7	4.7	4.1	0.848 ± 0.031	0.00764 ± 0.00215
*atp8*	159	11	14.2	8.8	7.5	0.820 ± 0.049	0.01424 ± 0.00306
*atp6*	663	15	21.1	5.6	4.7	0.899 ± 0.027	0.00909 ± 0.00258
*cox3*	778	14	25.8	3.9	3.2	0.888 ± 0.034	0.00579 ± 0.00135
*nad3*	354	9	18.2	5.9	5.4	0.695 ± 0.073	0.00965 ± 0.00246
*nad1*	939	16	22.9	5.6	5.2	0.935 ± 0.016	0.00991 ± 0.00241
*nad5*	1,650	27	19.9	5	4	0.976 ± 0.011	0.01416 ± 0.00195
*nad4*	1,326	28	23.8	6.8	5.6	0.961 ± 0.019	0.01656 ± 0.00232
*nad4l*	276	11	18.4	4.7	3.6	0.724 ± 0.071	0.01026 ± 0.00161
*nad6*	430	13	20.2	7.7	5.8	0.820 ± 0.050	0.01652 ± 0.00300
*cytb*	1,077	21	26	6.6	5.1	0.948 ± 0.017	0.01538 ± 0.00265
13 protein-coding genes	10,817	40	23.5	5.9	4.8	0.999 ± 0.006	0.01261 ± 0.00167

SD, standard deviation.

In the MCC phylogenetic tree for *A. variegatum*, it was evident that sequences derived from ticks collected in Malawi formed distinct clusters, separate from those obtained in Uganda, except for one sample MW-425 (S1 Fig). Similarly, most of the samples from Uganda formed distinct clusters except three samples, UG-D1, UG-D9, and UG-T1A (S1 Fig). The MJN analysis showed a similar pattern to the distribution observed in the phylogenetic analysis: samples from Malawi were separate from those collected in Uganda, with only sample MW-425 from Malawi clustered with those from Uganda, while samples UG-D1, UG-D9, and UG-T1A from Uganda clustered with those from Malawi (S2 Fig). These findings support geographical sub-structuring.

### 3.2. Tick mitogenome population structure

**Fig 2:**
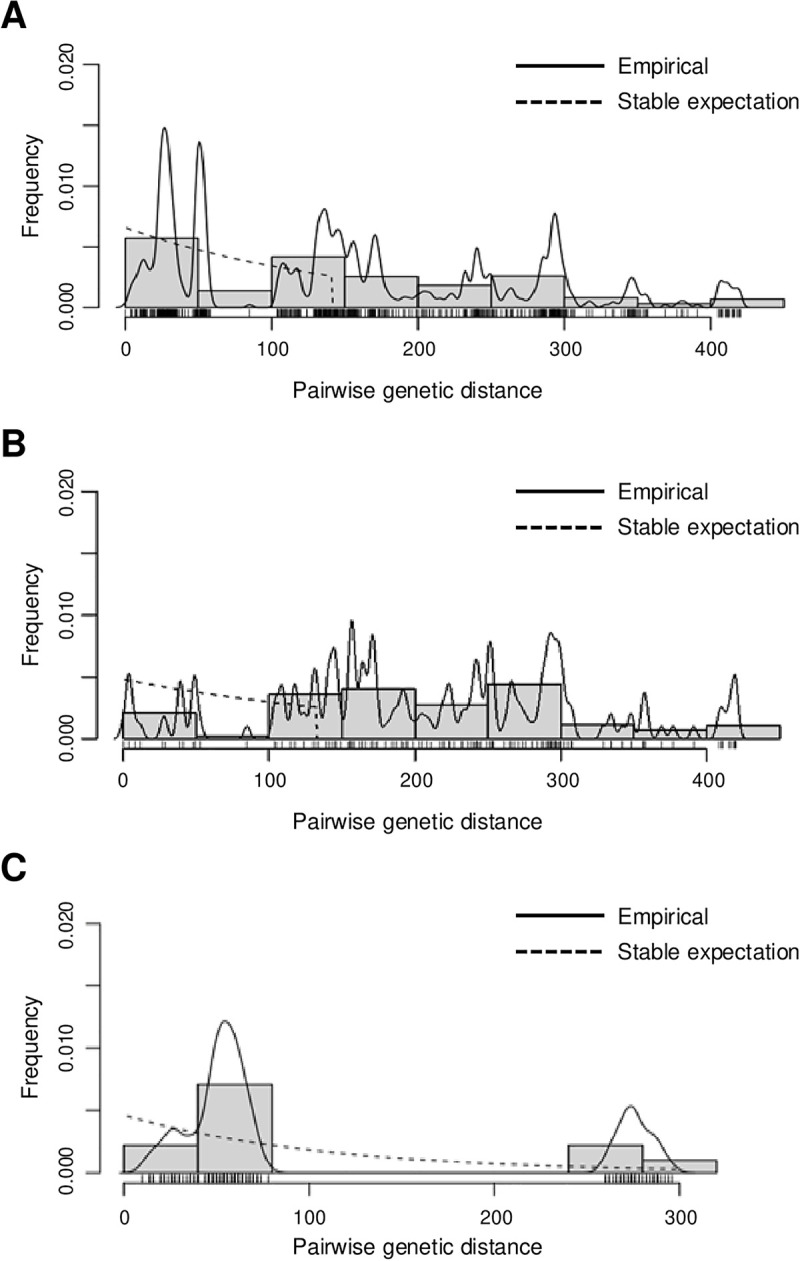
Mismatch distribution pattern for *Amblyomma variegatum* from Malawi and Uganda based on the 15 concatenated mitochondrial gene sequences. Mismatch distribution pattern for *A. variegatum* sequences from (A) Malawi and Uganda, (B) Malawi, and (C) Uganda. The x-axis shows the number of pairwise differences (genetic distance) between pairs of sequences and the y-axis shows their frequency. Solid histograms illustrate the observed frequencies. The solid black line indicates the simulated mismatch distributions expected under demographic expansion and the dotted black line indicates those expected under spatial expansion.

Results of the mismatch analysis based on pairwise nucleotide differences between 41 nucleotide sequences from Malawi (n = 20) and Uganda (n = 21) revealed that the shape of the mismatch distributions is far from a unimodal distribution when analyzing either sequences of *A. variegatum* from Malawi or Uganda (Fig 2). This indicates that the demographic history of *A. variegatum* in both Malawi and Uganda is more complex and likely involves multiple demographic events or population substructures. Specifically, in sequences from Uganda, a bimodal distribution implies that there have been at least two significant events in the population’s history that have led to the observed genetic diversity (Fig 2).

To investigate the genetic variance among and within the populations, the AMOVA showed that variation was mainly within the populations (74%) rather than among the populations (26%), as shown in Table 3. Our statistical power analysis yielded an empirical *p*-value of <0.001 and an estimated statistical power of 0.998, indicating that our sampling scheme had sufficient sensitivity to detect moderate to high levels of population differentiation (S2 Table). A significant negative Tajima’s *D* value suggests positive selection, or exhibiting a sudden population expansion after a recent bottleneck. A significant positive Tajima’s *D* value means increases in average pairwise genetic diversity in a population, indicating a balancing selection model or a population contraction event such as population subdivision. Non-significant and near-zero Tajima’s *D* values indicate a constant population size. In both the Malawi and Uganda populations of *A. variegatum*, we observed non-significant positive and negative values, respectively, at *P* <0.05 (Table 4).

**Table 3:** Analysis of Molecular Variance (AMOVA) for *Amblyomma variegatum* from Malawi and Uganda.

Source of variation	Sum of squares	Variance components	Percentage variation	*P*-value
Among populations	611.34	26.19643	25.98111 (Fst %)	<0.05
Within population	2910.635	74.63232	74.01889	<0.05
Total	3521.975	100.82875		

**Table 4:** Neutrality test values of *Amblyomma variegatum* from Malawi and Uganda based on complete mitogenomes.

Statistics		Malawi	Uganda	Mean	SD
Tajima’s *D*	Sample size	20	21	20.5	0.707
	S	651	284	467.5	259.508
	Pi	236.574	66.224	151.399	120.456
	Tajima’s *D*	0.955	−1.067	−0.056	1.429
	Tajima’s *D* *p*-value	0.878	0.135	0.507	0.526
Fu’s *Fs*	No. of allelles	20	21	20.5	0.707
	Theta_Pi	236.574	66.224	151.399	120.456
	Exp. no. of alleles	19.238	18.361	18.8	0.62
	Fu’s *Fs*	−0.17	−2.829	−1.5	1.88
	Fu’s *Fs p*-value	0.279	0.068	0.173	0.149

S, singleton sites; Pi, parsimony informative sites; SD, standard deviation.

### 3.3. Rickettsia genotyping and population structure

All the A. vari*egatum* samples from Malawi (n = 20) and Uganda (n = 21) were positive for *R. africae* by real-time PCR. The conventional PCR assays used in MLST of *R. africae* successfully amplified and sequenced six target genes, *gltA, htrA*, *ompA*, *ompB*, *sca4*, and 16S rDNA. The percentages of variable sites in the obtained sequences in these genes were 13%, 2%, 6%, 1%, 2%, and 2% for the *gltA*, *htrA*, *ompA*, *ompB*, *sca4*, and 16S rDNA, respectively.

The AMOVA revealed that the majority of variation occurred within populations (94.33%), while only a small portion was observed between populations (5.67%) as shown in Table 5. Our statistical power analysis yielded an empirical *p*-value of 0.058 and an estimated statistical power of 0.942, indicating that our sampling scheme had sufficient sensitivity to detect subtle but biologically meaningful levels of population differentiation in *R. africae* (S2 Table).The population genetic indices for *R. africae* for Malawi were 0.716 and −10.012, for Tajima’s *D* and Fu’s *Fs* statistic, respectively; while for Uganda, they were −0.827 and −8.096, for Tajima’s *D* and Fu’s *Fs* statistic, respectively (Table 6). The Tajima’s *D* and Fu’s *Fs* statistics for both populations were non-significant at *P* <0.05.

**Table 5:** Analysis of Molecular Variance (AMOVA) for *Rickettsia africae* from Malawi and Uganda.

Source of variation	Sum of squares	Variance components	Percentage variation	*P*-value
Among populations	18.716	0.5042	5.67164 (Fst %)	<0.05
Within population	327.04	8.38565	94.32836	<0.05
Total	345.756	8.88985		

**Table 6:** Neutrality test values of *Rickettsia africae* from Malawi and Uganda based on the concatenated six gene sequences.

Statistics		Malawi	Uganda	Mean	SD
Tajima’s *D*	Sample size	20	21	20.5	0.707
	S	58	58	74	22.627
	Pi	13.458	19.919	16.688	4.569
	Tajima’s *D*	0.716	−0.827	−0.771	0.079
	Tajima’s *D* *p*-value	0.248	0.21	0.229	0.0268
Fu’s *Fs*	No. of alleles	20	21	20.5	0.707
	Theta_Pi	13.458	19.919	16.688	4.569
	Exp. no. of alleles	12.56	14.6	13.58	1.442
	Fu’s *Fs*	−10.012	−8.096	−9.054	1.355
	Fu’s *Fs p*-value	0.001	0.005	0.003	0.003

S, singleton sites; Pi, parsimony informative sites; SD, standard deviation.

The phylogenetic analysis showed no evidence of geographical population sub-structuring in *R. africae*, as observed in *A. variegatum*, with sequences from Malawi and Uganda clustering together within the same clades (S3 Fig). This suggests that the *R. africae* is highly conserved in both populations and that they are closely related to each other.

### 3.4. Co-phylogeny of *Amblyomma variegatum* and *Rickettsia africae*

**Fig 3:**
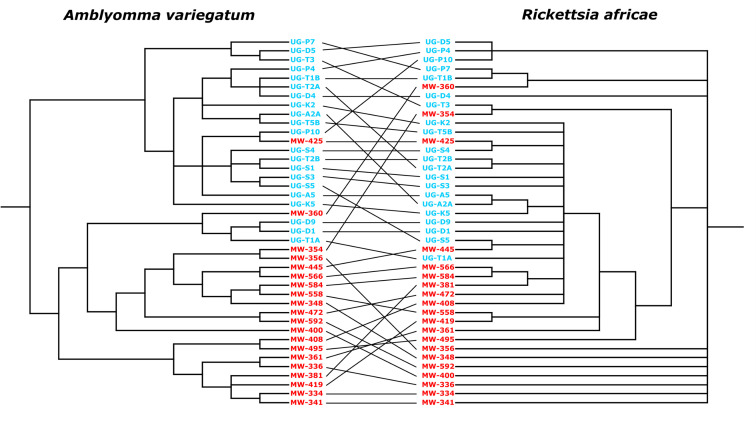
Tanglegram of the phylogeny of *Amblyomma variegatum* (left) and *Rickettsia africae* (right).

**Fig 4:**
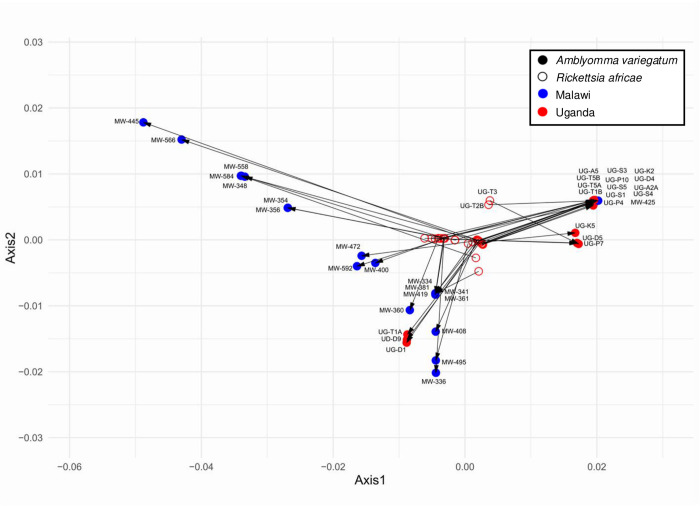
Procrustean superimposition plot of vector-pathogen associations based on their principal coordinate axes. Vector (*Amblyomma variegatum*) coordinates are shown as solid circles, and pathogen (*Rickettsia africae*) coordinates are shown as hollow circles, with arrows pointing from each pathogen to its corresponding vector. Samples from Malawi are shown in blue and samples from Uganda are shown in red. The X-axis and Y-axis represent the first two principal coordinate axes derived from the phylogenetic distance matrices, capturing the primary patterns of variation in vector and pathogen relationships. Arrows indicate the strength of vector-pathogen co-structure, with shorter arrows indicating closer congruence between vector and pathogen datasets. The m² value was 0.216, but the *p*-value was 0.064, indicating a nonsignificant association between vector and pathogen.

The tanglegram revealed very little co-cladogenesis between *R. africae* and *A. variegatum* (Fig 3). The overall phylogenetic patterns of the vector and pathogen trees were largely incongruent. The Procrustes superimposition plot showed long arrows between the vector and pathogen coordinates, indicating a weak association between the two (Fig 4). The m² value was 0.216, and the *p*-value was 0.064, suggesting a statistically nonsignificant relationship between the vector and pathogen. *P*-values were derived from 1,000 permutations of the vector-pathogen association matrix, generating randomized m² values (S4 Fig).

## 4. Discussion

The analysis of the complete mitogenome of *A. variegatum* showed that the A + T and G + C contents were similar to those reported in other Metastriata ticks at 76.8% and 23.2%, respectively [65]. Among the PCGs, the genes with a high proportion of polymorphic sites were *atp8*, *nad6*, *nad2*, and *nad4* at 8.8%, 7.7%, 7.4%, and 6.8%, respectively. These highly polymorphic genes are good genetic markers to be used for intra-species genotyping and characterization of *A. variegatum.* The phylogenetic analysis revealed that the mitogenomes clustered into two clades: clade A, which comprised predominantly of samples from Malawi, except for three samples from Uganda; and clade B, which was comprised predominantly of samples from Uganda, with only one sample from Malawi. This has shown population sub-structuring of the *A. variegatum* mitogenomes.

The population genetic analysis of the mitogenomes of *A. variegatum* samples from Malawi has shown that the variation is greater within populations than among populations. Further, both the Tajima’s *D* and Fu’s *Fs* statistics analyses yielded non-significant values, supporting the hypothesis of stable populations. The mismatch distribution indicates that the distribution of the two populations of *A. variegatum* are far from uniform. Moreover, the population structure analysis revealed that neither population exhibited a unimodal distribution. This phenomenon was also exhibited in other *Amblyomma* species, such as *Amblyomma testudinarium* samples from Japan and Myanmar [68]. This was very clear in the Ugandan samples which showed a bimodal distribution. Differences in weather patterns between the two countries may explain the bimodal distribution observed in Uganda. Unlike Malawi, which has a relatively uniform climate, Uganda experiences seasonal variations, particularly in the central and southern regions with an equatorial climate [69]. These areas have two rainy seasons (March–May and September–November) and two dry seasons (January–February and June–August) [69]. In contrast, northern Uganda has one dry season from December to February and one rainy season from April to October. Moreover, Uganda has consistently high temperatures ranging from 25^o^C to 29^o^C which provide optimum conditions for the thriving of *A. variegatum* throughout the year [3]. The different weather conditions in Uganda may exert increased selective pressure on *A. variegatum*, driving genetic adaptations to different climatic conditions. In contrast, Malawi’s more stable climate, with a single rainy season (November–April) and a single dry season (May–October) across the country, may impose less environmental pressure for such genetic changes [70]. There have been recent reports of *A. variegatum* detections in European countries, such as Italy [1] and France [2] as well as in regions outside Africa such as the Caribbean islands [3,4] and the Middle East [9,71]. These findings support the hypothesis that *A. variegatum*’s ecological plasticity contributes to its spread beyond its native range [1].

The observed 100% prevalence of *R. africae* in the *A. variegatum* samples used in this study supports the endemicity of this pathogen in the regions. This underscores the need to consider this neglected tick-borne rickettsiosis as a key pathogen when evaluating individuals with reported tick bites who exhibit malaria-like symptoms [8,15,33]. The phylogenetic analysis did not separate the sequences from Malawi from those from Uganda, which indicates that the strains of *R. africae* circulating in both countries are closely related and may share a common ancestor [61]. Moreover, the population genetic analysis using Tajima’s *D* and Fu’s *Fs* statistics did not yield any statistically significant values, indicating that the *R. africae* populations in the two countries are stable [61,68].

*Rickettsia africae* is transmitted vertically within its principal tick vectors, *A. hebraeum* and *A. variegatum,* and has been reported to have high infection rates of up to 100% [72]. Vertical transmission of *R. africae* in *Amblyomma* ticks has facilitated its successful adaptation and establishment in tick vectors than any other *Rickettsia* species [73]. This is supported by the most important observation that *R. africae* infection does not negatively affect the fitness of the vector *Amblyomma* ticks [23]. However, the limited co-cladogenesis observed between the phylogenetic trees of *R. africae* and *A. variegatum* does not support the hypothesis that *R. africae* is predominantly vertically transmitted among *A. variegatum* populations, which would be reflected by congruent vector–pathogen phylogenies. The horizontal transmission may have contributed to the loss of strict co-cladogenesis between *R. africae* and the vector tick *A. variegatum* observed in this study. This has also enabled *R. africae* to infect humans, establishing itself as a zoonotic species. *Rickettsia africae* is one of the most common spotted fever group (SFG) rickettsias that are diagnosed in humans based on seroprevalence and incidence rates [22,23,27].

Horizontal transmission potential may indicate its ability to be transmitted to and maintained in other tick species. Several studies reported *R. africae* in non-*Amblyomma* species such as *Rhipicephalus appendiculatus* and *Rhipicephalus microplus* [24,25,27]. These findings also highlight the potential of *R. africae* to adapt to different tick vectors, implying a potential expansion of its vectoral range, which could complicate efforts to control it as a human pathogen [30]. The involvement of humans or other vertebrate species in the transmission and maintenance cycle of *R. africae* adds versatility to its life cycle. Unlike simple tick-to-tick transmission, the tick-vertebrate-tick pathway allows the bacterium to persist and spread across different regions, making it more difficult to control [39–41]. This vertebrate-mediated transmission is further facilitated by the infection of *R. africae* in a wide range of vertebrate species and by the frequent movement of humans and livestock across long distances in many parts of Africa. Together, these factors significantly increase the risk of pathogen spread and pose a major public health concern [43].

In this study, the symbiotic relationship between *A. variegatum* and *R. africae* was found to be relatively unstable, as shown by the lack of strict co-cladogenesis in their phylogenetic trees and supported by the PACo analysis. This observation aligns with that of Duron et al. [73], who reported that obligate symbioses are unstable in ticks. Similarly, the absence of strict co-cladogenesis observed in this study supports earlier results by Weinert et al. [74], who noted that unlike many mutualistic symbionts whose evolutionary histories mirror those of their hosts (indicating co-evolution) *Rickettsia* species often lack such phylogenetic congruence. This instability may result from the ease with which *Rickettsia* symbioses are lost over time, leading to flexible evolutionary relationships. Such flexibility may facilitate transitions from symbionts to pathogens, enabling infections in invertebrate species [75,76]. The coexistence of both vertical and horizontal transmission modes in *R. africae* may contribute to its unstable association with its vector tick, a phenomenon also reported for *Coxiella*-like endosymbionts (CLE) [77]. Vertical transmission is essential for the persistence of the symbiont within the primary vector tick population, while horizontal transmission enables it to acquire pathogenic potential and infect other vertebrate species [76].

*Amblyomma variegatum* and its endosymbiont *R. africae* have been reported to expand their geographical distribution and vertebrate host range by unlocking new niches in the Caribbean islands and some European countries [1–4]. Previous studies reported the expansion of ecological range and diversification among heritable symbionts such as *Buchnera aphidicola* in aphids and CLE in ticks [30,73]. The implication of vector-symbiont range expansion, as exhibited by *A. variegatum* and *R. africae*, ensures that the symbionts can acquire genes responsible for environmental adaptation, which are important for expansion of the range and host maintenance [73,77,78]. This maintenance will cause *R. africae* to remain an important pathogen; wherever *A. variegatum* is present, people are put at risk of ATBF. Therefore, a multi-sectoral coordinated (One Health) approach to vector surveillance and pathogen maintenance is required to establish a broader, comprehensive ecological and epidemiological data management system.

It is important to note that the mitogenome only reflects the maternal inheritance and does not represent the recombination and gene flow patterns of the nuclear genome. Therefore, genetic variation from the nuclear genome, which may influence traits like vector competence or tick–pathogen interactions, is not captured. It is recommended that future studies integrate nuclear and mitochondrial markers as well as microbiome data and functional genomics for a more robust and complete understanding of the transmission dynamics and vector-pathogen co-evolution. More research is required to determine how *A. variegatum* microbiomes affect the fitness of the tick and its capacity for vertical transmission of *R. africae* to other tick species and vertebrate species like humans. Other limitations in this study include the limited sample size and the limited geographical coverage of the sampling sites. There is also a need to compare the mitogenome of *A. variegatum* from different animal species, including wildlife, to understand their population genetics and host relationships. Studies focusing on the co-phylogeny of ticks and their endosymbionts are warranted to validate the findings of this study.

In conclusion, this study successfully reports the complete mitogenomes of *A. variegatum* for the first time and tested the hypothesis that *R. africae* is predominantly transmitted vertically within *A. variegatum* populations using a mitogenomic approach. The generated data provides a valuable resource for researchers studying tick phylogeny and evolutionary genetics. Finally, ATBF should be considered in the differential diagnosis of patients presenting with malaria-like symptoms, particularly those with a history of travel to endemic regions or potential exposure to tick bites in such areas.

## Supporting information

S1 Table Mitochondrial genome organization of *Amblyomma variegatum.* (DOCX)

S2 Table Statistical power estimation of Analysis of Molecular Variance (AMOVA) between Malawi and Uganda populations of *Amblyomma variegatum* and *Rickettsia africae.* (DOCX)

S1 Fig Bayesian phylogenetic Maximum Clade Credibility (MCC) tree of 41 *Amblyomma variegatum* sequences based on entire mitochondrial genomes. (DOCX)

S2 Fig Median-joining network (MJN) for *Amblyomma variegatum* from Malawi and Uganda based on complete mitogenomes. (DOCX)

S3 Fig Bayesian phylogenetic Maximum Clade Credibility (MCC) tree of *Rickettsia africae* sequences based on the six concatenated genes. (DOCX)

S4 Fig Distribution of randomized m² values from procrustean analysis. (DOCX)

S1 Data Word document for the PLOS NTDs striking image. (DOCX)
