# Supplementary material for: Mitogenomics of the tropical bont tick Amblyomma variegatum reveals vertical and horizontal transmission of Rickettsia africae
Source: PLoS Negl Trop Dis. 2025 Oct 21;19(10):e0013610. doi: 10.1371/journal.pntd.0013610 (PMC12551961; doi:10.1371/journal.pntd.0013610)
Supplement: S1 Table — (DOCX) [file pntd.0013610.s001.docx]

**Table S1. Mitogenome organization of *Amblyomma variegatum*.**

| **Gene** | **Strand** | **Position** | **Length (bp)** | **Intergenic spacer*** | **Start codon** | **Stop codon** | **Anticodon** |
| --- | --- | --- | --- | --- | --- | --- | --- |
| tRNA-Met | + | 2–63 | 62 | 1 |  |  | ATG |
| ND2 | + | 64–1,026 | 963 | 0 | ATA | TAA |  |
| tRNA-Trp | + | 1,025–1,085 | 61 | -2 |  |  | TGA |
| tRNA-Tyr | - | 1,084–1,146 | 63 | -2 |  |  | TAC |
| COX1 | + | 1,139–2,677 | 1539 | -8 | ATT | TAA |  |
| COX2 | + | 2,681–3,355 | 675 | 3 | ATG | TAA |  |
| tRNA-Lys | + | 3,357–3,423 | 67 | 1 |  |  | AAG |
| tRNA-Asp | + | 3,423–3,485 | 67 | 0 |  |  | GAC |
| ATP8 | + | 3,485–3,643 | 159 | 0 | ATT | TAA |  |
| ATP6 | + | 3,638–4,300 | 663 | -6 | ATG | TAA |  |
| COX3 | + | 4,304–5,081 | 778 | 3 | ATG | TAA |  |
| tRNA-Gly | + | 5,082–5,141 | 60 | 0 |  |  | GGA |
| ND3 | + | 5,142–5,483 | 342 | 0 | ATT | TAA |  |
| tRNA-Ala | + | 5,483–5,544 | 62 | -1 |  |  | GCA |
| tRNA-Arg | + | 5,547–5,608 | 62 | 2 |  |  | CGA |
| tRNA-Glu | + | 5,609–5,669 | 61 | 0 |  |  | AAC |
| tRNA-Ser1 | + | 5,670–5,723 | 54 | 0 |  |  | AGA |
| tRNA-Glu | + | 5,724–5,785 | 61 | 0 |  |  | GAA |
| ND1 | - | 5,778–6,716 | 939 | -7 | ATT | TAA |  |
| tRNA-Leu2 | - | 6,718–6,779 | 62 | 1 |  |  | TTA |
| 16S rRNA | - | 6,779–7,982 | 1204 | -1 |  |  |  |
| tRNA-Val | - | 7,983–8,043 | 61 | 0 |  |  | GTA |
| 12S rRNA | - | 8,044–8,740 | 697 | 0 |  |  |  |
| CR1 | + | 8,741–9,044 | 304 | 0 |  |  |  |
| tRNA-Ile | + | 9,045–9,108 | 63 | 0 |  |  | ATC |
| tRNA-Gln | - | 9,111–9,178 | 68 | 2 |  |  | CAA |
| tRNA-Phe | - | 9,179–9,239 | 61 | 0 |  |  | TTC |
| NAD5 | - | 9,240–10,889 | 1650 | 0 | ATT | TAA |  |
| tRNA-His | - | 10,890–10,951 | 62 | 0 |  |  | CAC |
| NAD4 | - | 10,951–12,276 | 1326 | -1 | ATG | TAG |  |
| NAD4L | - | 12,270–12,546 | 276 | -7 | ATG | TAA |  |
| tRNA-Thr | + | 12,548–12,609 | 62 | 1 |  |  | ACA |
| tRNA-Pro | - | 12,614–12,680 | 67 | 4 |  |  | CCA |
| NAD6 | + | 12,687–13,116 | 430 | 6 | ATT | TGA |  |
| CYTB | + | 13,117–14,193 | 1077 | 0 | ATG | TAG |  |
| tRNA-Ser2 | + | 14,193–14,255 | 63 | -1 |  |  | TCA |
| tRNA-Leu1 | - | 14,255–14,315 | 61 | -1 |  |  | CTA |
| CR2 | + | 14,316–14,584 | 269 | 0 |  |  |  |
| tRNA-Cys | + | 14,585–14,637 | 53 | 0 |  |  | TGC |

* Negative values in the intergenic spacer indicate overlap between genes.
