# Supplementary material for: Mitogenomics of the tropical bont tick Amblyomma variegatum reveals vertical and horizontal transmission of Rickettsia africae
Source: PLoS Negl Trop Dis. 2025 Oct 21;19(10):e0013610. doi: 10.1371/journal.pntd.0013610 (PMC12551961; doi:10.1371/journal.pntd.0013610)
Supplement: S2 Table — (DOCX) [file pntd.0013610.s002.docx]

**Table S2. Statistical power estimation of Analysis of Molecular Variance (AMOVA) between Malawi and Uganda populations of *Amblyomma variegatum* and *Rickettsia africae*.**

| Species | Observed ΦST | Permutations | P-value | Statistical power |
| --- | --- | --- | --- | --- |
| *Amblyomma variegatum* | 0.3111 | 1,000 | <0.001 | 0.998 |
| *Rickettsia africae* | 0.0608 | 1,000 | 0.058 | 0.942 |
