# Supplementary material for: Mitogenomics of the tropical bont tick Amblyomma variegatum reveals vertical and horizontal transmission of Rickettsia africae
Source: PLoS Negl Trop Dis. 2025 Oct 21;19(10):e0013610. doi: 10.1371/journal.pntd.0013610 (PMC12551961; doi:10.1371/journal.pntd.0013610)
Supplement: S1 Fig — (DOCX) [file pntd.0013610.s003.docx]

*
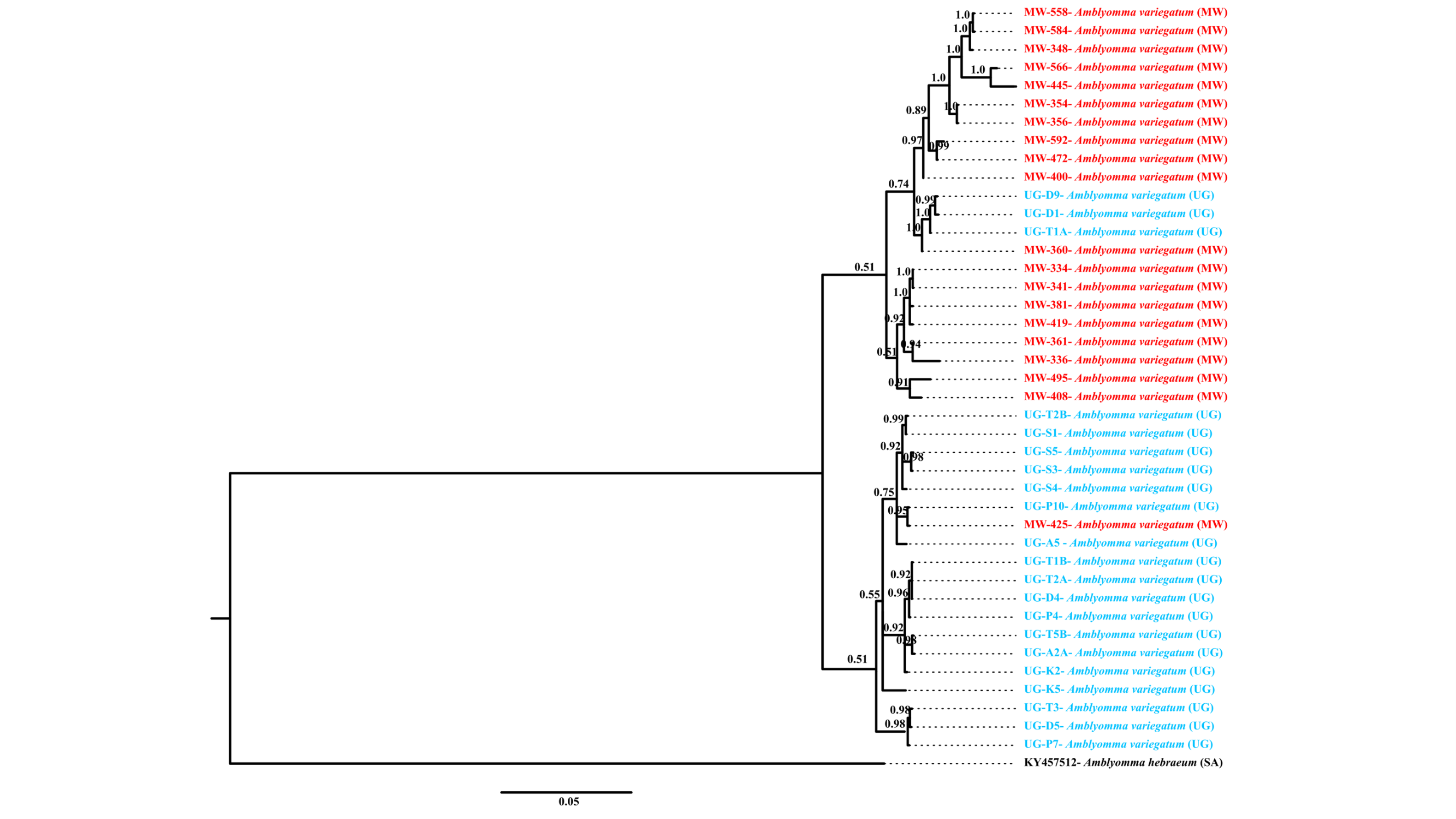
*

**Figure S1. Bayesian phylogenetic Maximum Clade Credibility (MCC) tree of 41 *Amblyomma variegatum* sequences based on entire mitochondrial genomes.** Sequences of *A. variegatum* from Malawi and Uganda are highlighted in red and blue, respectively and *A. hebraeum* obtained from the GenBank NCBI database was set as the outgroup. The alphanumeric code preceding the tick species name serves as the GenBank accession number or sample ID. Abbreviations in parentheses following the tick species name refer to the country of origin: MW for Malawi, SA for South Africa, and UG for Uganda.
