# Supplementary material for: Mitogenomics of the tropical bont tick Amblyomma variegatum reveals vertical and horizontal transmission of Rickettsia africae
Source: PLoS Negl Trop Dis. 2025 Oct 21;19(10):e0013610. doi: 10.1371/journal.pntd.0013610 (PMC12551961; doi:10.1371/journal.pntd.0013610)
Supplement: S2 Fig — (DOCX) [file pntd.0013610.s004.docx]

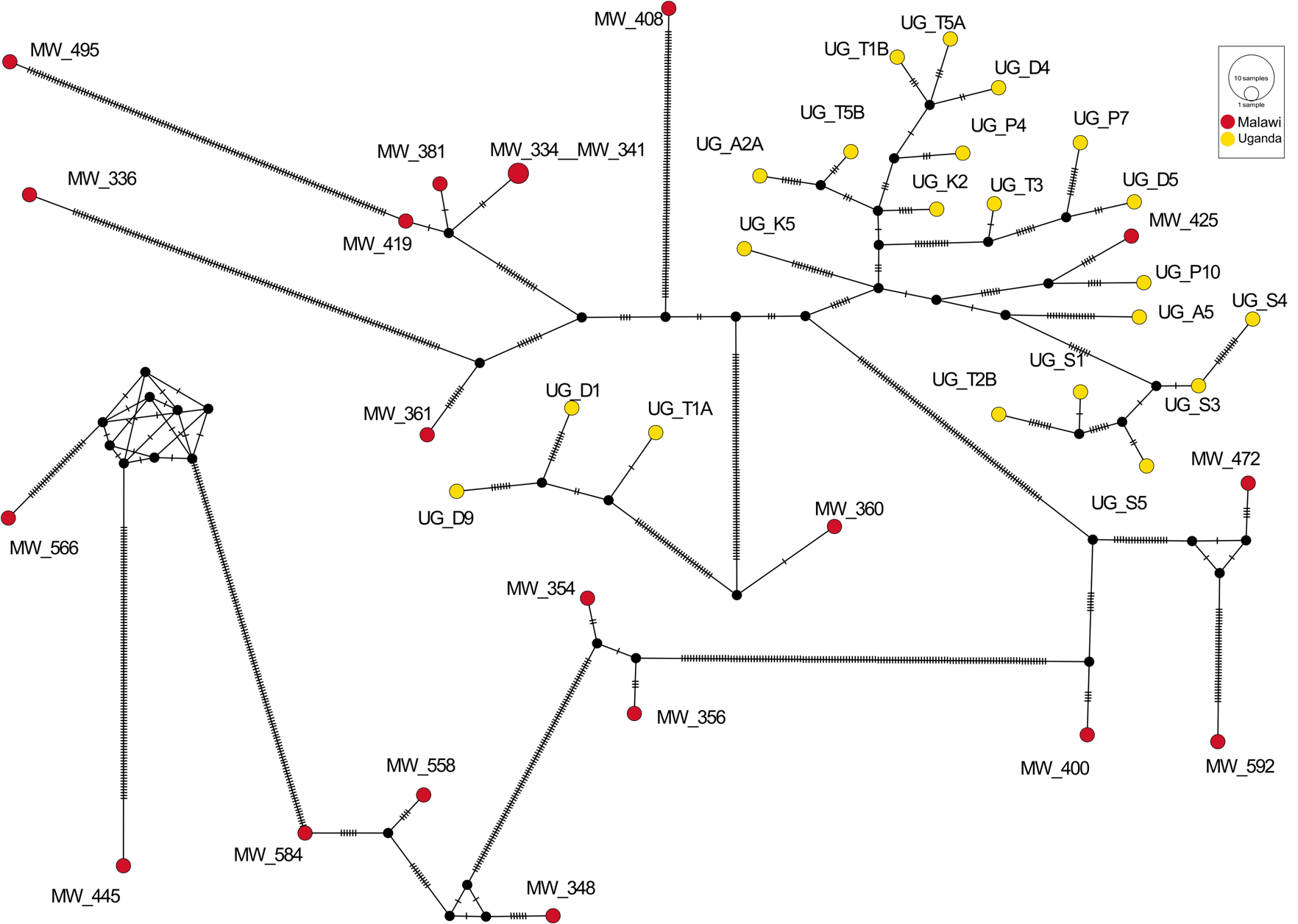


**Figure S2. Median-joining network (MJN) for *Amblyomma variegatum* from Malawi and Uganda based on complete mitogenomes.** MW, Malawi; UG, Uganda.
