# Supplementary material for: Mitogenomics of the tropical bont tick Amblyomma variegatum reveals vertical and horizontal transmission of Rickettsia africae
Source: PLoS Negl Trop Dis. 2025 Oct 21;19(10):e0013610. doi: 10.1371/journal.pntd.0013610 (PMC12551961; doi:10.1371/journal.pntd.0013610)
Supplement: S3 Fig — (DOCX) [file pntd.0013610.s005.docx]

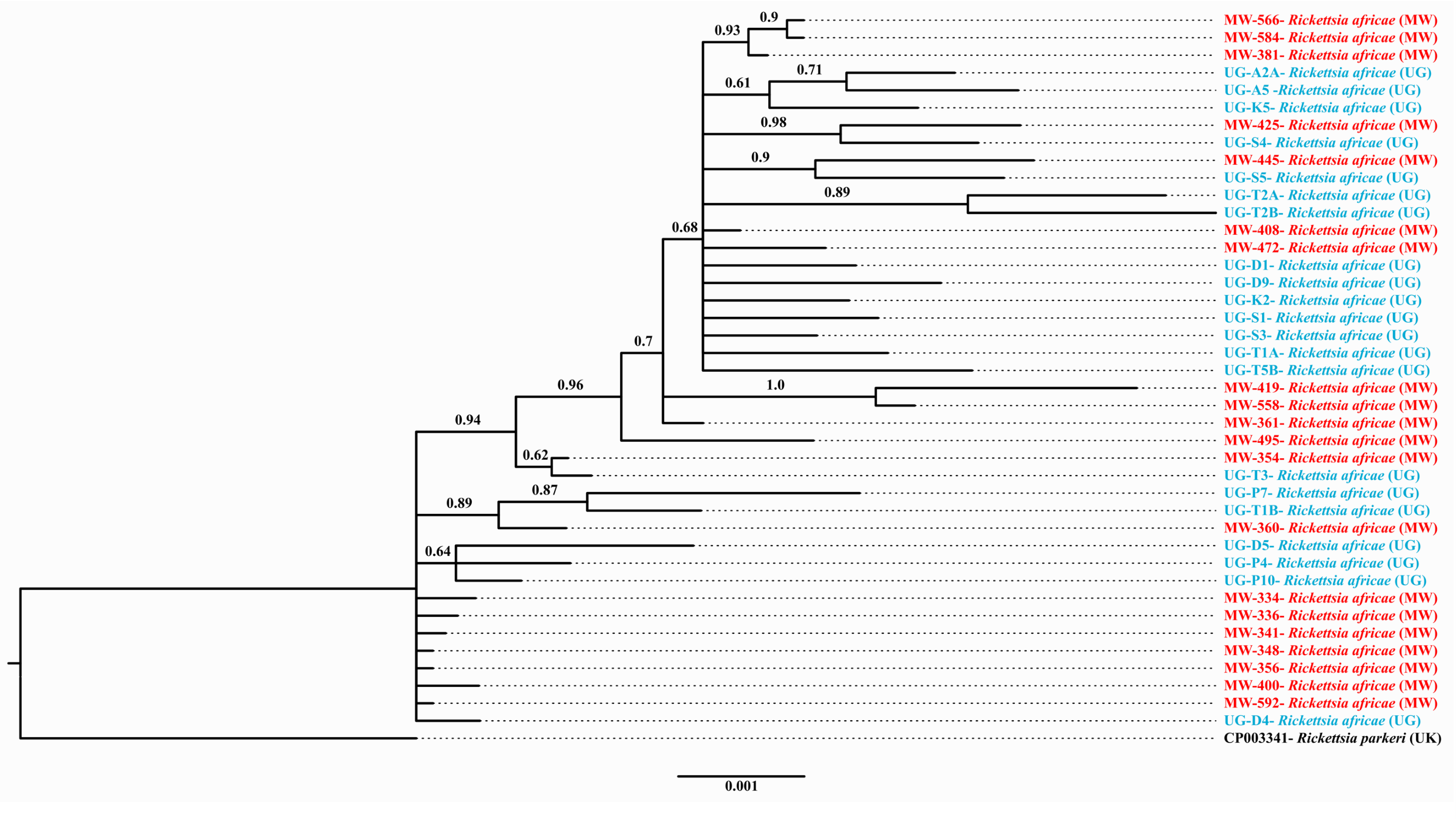


**Figure S3. Bayesian phylogenetic Maximum Clade Credibility (MCC) tree of *Rickettsia africae* sequences based on the six concatenated genes.** Sequences of *R. africae* from Malawi and Uganda are highlighted in red and blue, respectively. *Rickettsia parkeri* (CP_003341), obtained from the GenBank NCBI database, was set as the outgroup. The alphanumeric code preceding the species name serves as the GenBank accession number or sample ID. Abbreviations in parentheses following the species name refer to the country of origin: MW for Malawi, UG for Uganda, and UK for the United Kingdom.
