# Supplementary material for: Mitogenomics of the tropical bont tick Amblyomma variegatum reveals vertical and horizontal transmission of Rickettsia africae
Source: PLoS Negl Trop Dis. 2025 Oct 21;19(10):e0013610. doi: 10.1371/journal.pntd.0013610 (PMC12551961; doi:10.1371/journal.pntd.0013610)
Supplement: S4 Fig — (DOCX) [file pntd.0013610.s006.docx]

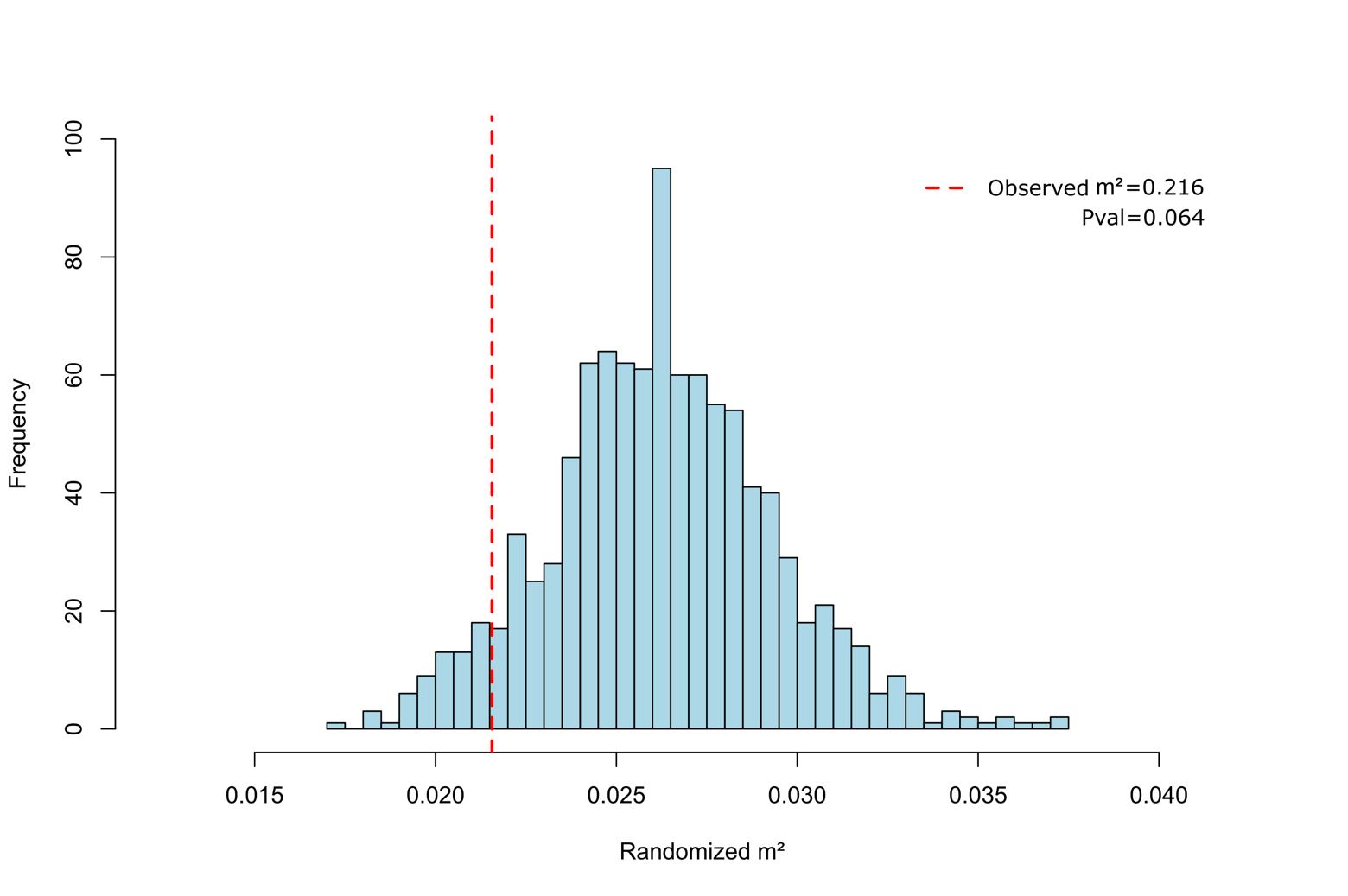


**Figure S4. Distribution of randomized m² values from procrustean analysis.**

The histogram depicts the distribution of randomized m² values generated from 1,000 permutations of the vector-pathogen association matrix. The x-axis represents the randomized m² values, while the y-axis indicates the frequency of each value across the permutations. The vertical dashed line denotes the observed m² value (0.216) from the original vector-pathogen associations. In this analysis, the observed m² was not significantly different from the randomized distribution (*P*-value = 0.064), indicating that the vector-pathogen associations do not show strong congruence beyond what could be expected by chance.
