# Supplementary material for: Mitogenomics of the tropical bont tick Amblyomma variegatum reveals vertical and horizontal transmission of Rickettsia africae
Source: PLoS Negl Trop Dis. 2025 Oct 21;19(10):e0013610. doi: 10.1371/journal.pntd.0013610 (PMC12551961; doi:10.1371/journal.pntd.0013610)
Supplement: S1 Data — (DOCX) [file pntd.0013610.s007.docx]

1)caption

The tropical bont tick (*Amblyomma variegatum*) is one of Africa's most beautiful parasites, but also one of its most persistent problems. It frequently bites humans and is a major vector of African tick-bite fever, caused by *Rickettsia africae*. Alongside its medical impacts, the tropical bont tick commonly infests livestock resulting in a range of livestock disease.

2)credit

Elisha Chatanga and Yuki Ohsugi

3)confirmation

We would like to confirm that this image can be published under the Creative Commons Attribution License (https://creativecommons.org/licenses/by/4.0/).
